# The Many Faces of Primary Aldosteronism and Cushing Syndrome: A Reflection of Adrenocortical Tumor Heterogeneity

**DOI:** 10.3389/fmed.2018.00054

**Published:** 2018-03-12

**Authors:** Ozgur Mete, Kai Duan

**Affiliations:** ^1^Department of Pathology, University Health Network, Toronto, ON, Canada; ^2^Department of Laboratory Medicine and Pathobiology, University of Toronto, Toronto, ON, Canada; ^3^Endocrine Oncology Site Group, Princess Margaret Cancer Centre, Toronto, ON, Canada

**Keywords:** adrenocortical adenoma, primary pigmented nodular adrenocortical disease, tumor heterogeneity, endocrinology, primary aldosteronism, Cushing syndrome

## Abstract

Adrenal cortical tumors constitute a heterogeneous group of neoplasms with distinct clinical, morphological, and molecular features. Recent discoveries of specific genotype–phenotype correlations in adrenal cortical adenomas have transformed our understanding of their respective endocrine syndromes. Indeed, a proportion of patients with primary aldosteronism are now known to harbor adrenal cortical adenomas with heterogeneous molecular alterations (*KCNJ5, ATP1A1, ATP2B3*, and *CACNA1D*) involving the calcium/calmodulin kinase signaling pathway. Several lines of evidence suggest that *KCNJ5*-mutant aldosterone-producing adenomas have distinct clinicopathological phenotype compared to those harboring *ATP1A1, ATP2B3*, and *CACNA1D* mutations. Benign adrenal cortical tumors presenting with Cushing syndrome often have diverse mutations (*PRKACA, PRKAR1A, GNAS, PDE11A*, and *PDE8B*) involving the cyclic AMP signaling pathway. In addition to cortisol-producing adenomas, bilateral micronodular adrenocortical disease and primary bilateral macronodular adrenal hyperplasia (PBMAH) have also expanded the spectrum of benign neoplasms causing adrenal Cushing disease. The recent discovery of inactivating *ARMC5* germline mutations in PBMAH has challenged the old belief that this disorder is mainly a sporadic disease. Emerging evidence suggests that PBMAH harbors multiple distinct clonal proliferations, reflecting the heterogeneous genomic landscape of this disease. Although most solitary adrenal cortical tumors are sporadic, there is an increasing recognition that inherited susceptibility syndromes may also play a role in their pathogenesis. This review highlights the molecular and morphological heterogeneity of benign adrenal cortical neoplasms, reflected in the diverse presentations of primary aldosteronism and adrenal Cushing syndrome.

## Introduction

The past decade has seen tremendous growth in our understanding of the clinical, molecular, and histopathologic characteristics of adrenal cortical neoplasia. Precise clinical and molecular studies have demonstrated that primary adrenal cortical malignancy is not a single disease entity. In fact, it is now well recognized that adrenal cortical carcinomas display a wide spectrum of clinical manifestations with heterogeneous molecular and histopathologic features, as well as distinctive proliferative biology, biomarker expression, and prognostic cluster profiles. While the distinction of carcinomas from adenomas is critical, the recent discovery of specific genotype–phenotype correlations in aldosterone- and glucocorticoid-producing benign adrenal cortical tumors has significant implications for patient management. This review provides an update on the newly described molecular histophenotypic correlates of benign adrenal cortical tumors, resulting in heterogeneous presentations of hyperaldosteronism and hypercortisolism.

## Heterogeneity in Aldosterone-Producing Benign Adrenal Cortical Neoplasms

### Clinical and Histopathological Heterogeneity

Since the first description by Conn in 1955, much has been learned regarding the clinical, pathological, and molecular properties of primary aldosteronism (1–4). Inappropriate or autonomous aldosterone secretion, often detected through serum aldosterone-to-renin ratio, is attributed to a group of adrenal cortical proliferations that includes adrenal cortical hyperplasia, adenoma, and rare carcinomas (5, 6). Currently, bilateral adrenal cortical hyperplasia is regarded as the most common cause of primary aldosteronism, often diagnosed in older patients with a slight male predilection (1–4, 7). Affected individuals with bilateral disease often present with less severe hypertension and more frequent normokalemia (1–4, 7). In contrast, those with unilateral disease (i.e., aldosterone-producing adenoma) generally have more severe hypertension and more frequent hypokalemia (1–4, 7). Adrenal venous sampling has emerged as an important diagnostic tool to help localize the source of aldosterone excess and to distinguish unilateral from bilateral adrenal disease. Since the latter is treated medically, aldosterone-producing tumors are much more commonly seen in adrenalectomy specimens taken for primary aldosteronism.

Most aldosterone-producing adenomas are composed of tumor cells rich in lipid contents. As a result, adenomas typically exhibit lower attenuation (<10 Hounsfield units) on unenhanced CT imaging studies (8, 9), and their cut surface often appear golden yellow (5, 6). The application of CYP11B2 (aldosterone synthase) immunohistochemistry has enhanced our understanding of CT-detected adrenal nodules, as these may not necessarily represent the source of aldosterone excess, even when ipsilateral lateralization is detected by adrenal vein sampling, likely because additional smaller/microscopic cortical proliferations may be responsible for the hormonal overproduction (10–12). To complicate this conundrum, zona glomerulosa (ZG) hyperplasia or multiple functional micronodules have also been described in patients with primary aldosteronism (11, 13, 14).

**Figure 1:**
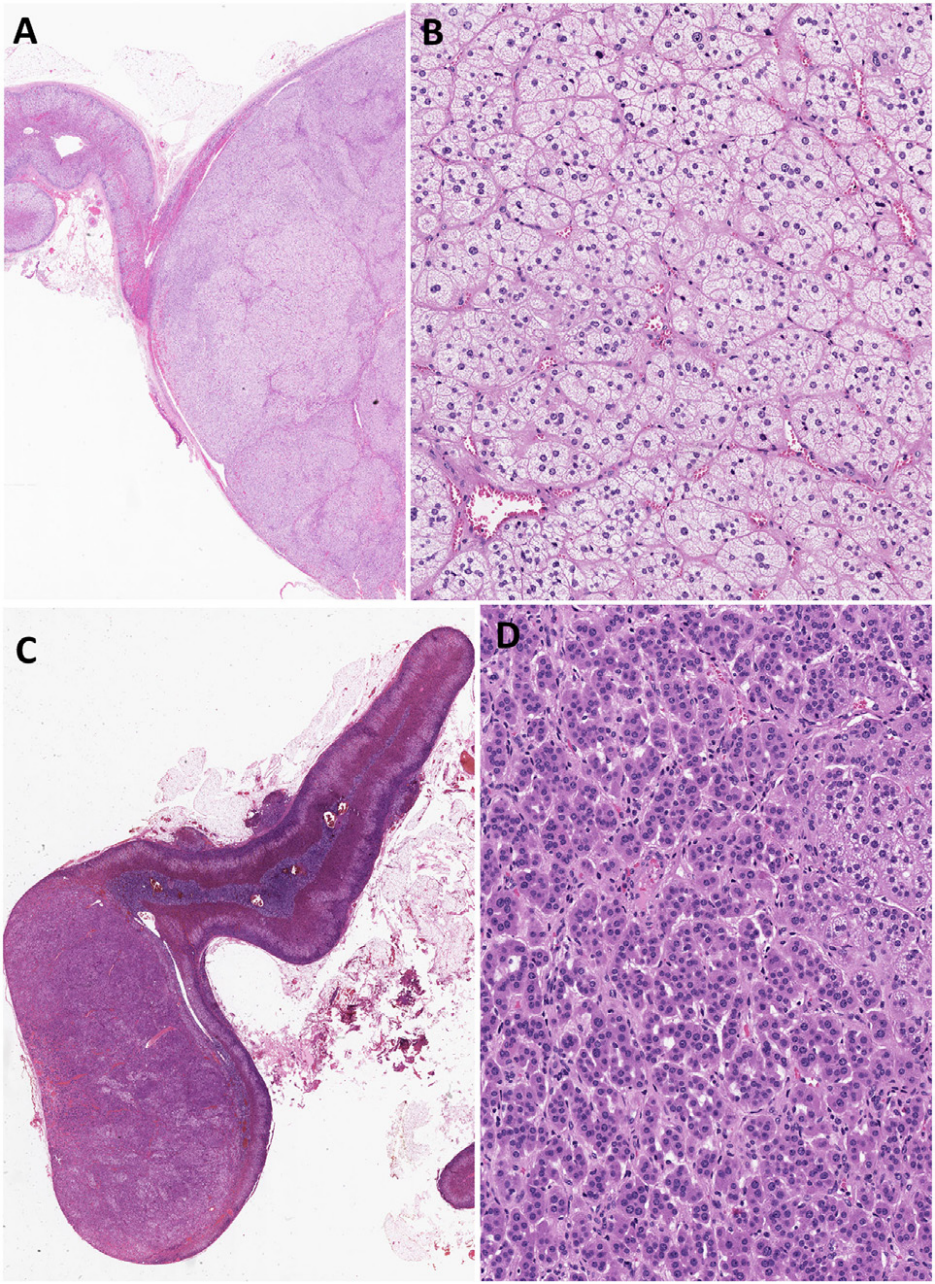
Heterogeneity in aldosterone-producing adrenocortical adenomas. The cytomorphological properties of an individual tumor can be heterogeneous due to variation in the composition of different cell types **(A–D)**. Most aldosterone-producing tumors are predominantly composed of clear cells with high lipid contents **(A,B)**. However, recent evidence suggests that variations in cytomorphological features exist due to the underlying molecular heterogeneity of these neoplasms. For instance, aldosterone-producing adenomas composed mainly of zona fasciculata-like clear cells **(A,B)** often harbor *KCNJ5* mutations. In contrast, tumors that are composed predominantly of compact cells typically show *ATP1A1, ATP2B3*, and *CACNA1D* molecular alterations **(C,D)**.

Most aldosterone-producing tumors are predominantly composed of clear cells with high lipid content. The neoplastic cells contain mitochondria with lamellar type or plate-like cristae. It should be noted that the cytomorphological details of an individual tumor can be heterogeneous due to variation in four distinct types of tumor cells that have been characterized in aldosterone-producing adenomas: (i) zona fasciculata (ZF)-like clear cells, (ii) ZG-like cells, (iii) zona-reticularis-like compact cells, and (iv) compact cells displaying overlapping cytomorphological features of both ZG- and fasciculata-like cells (5, 6) (Figure 1). Recent evidence suggests that the heterogeneous cytomorphological features of an aldosterone-producing adenoma may be related to its underlying genotype, reflecting different CYP11B2 (aldosterone synthase) and CYP11B1 (11β-hydroxylase that converts 11-deoxycortisol to cortisol) expression profile in the tumor (see below) (14–18). The presence of oncocytic cytomorphology also expands the histopathologic heterogeneity of aldosterone-producing adrenal cortical adenomas, including those with intracytoplasmic globular inclusions due to degenerate mitochondria (19).

### Molecular Characteristics and Pathogenesis

**Figure 2:**
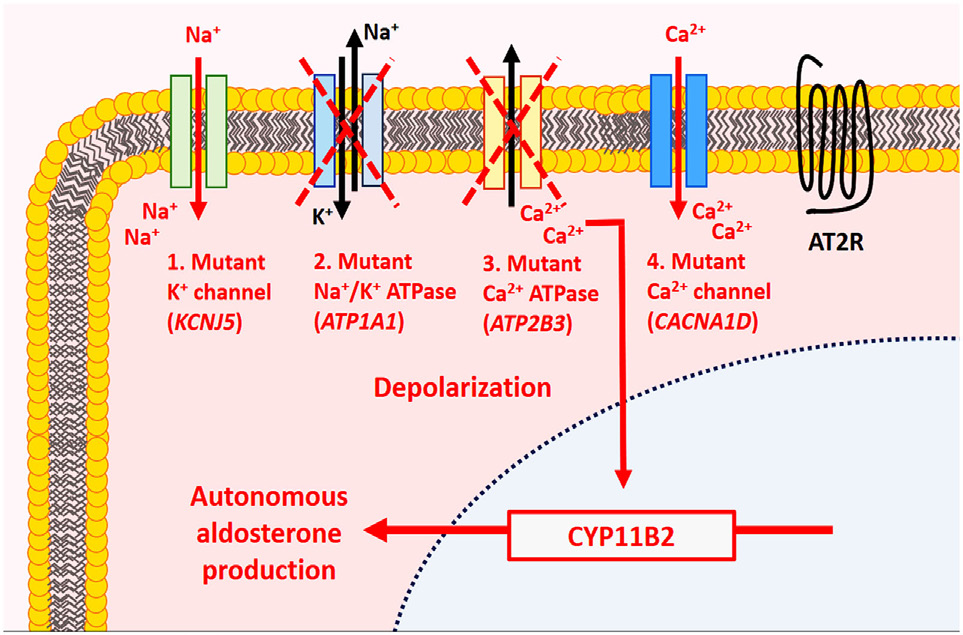
Molecular alterations in aldosterone-producing benign adrenal cortical neoplasms. In normal physiology, aldosterone production by adrenal zona glomerulosa (ZG) cells is mediated by the calcium/calmodulin signaling pathway. In the resting state, ZG cells are hyperpolarized, a process mainly mediated by K^+^ channels. Activation of the renin–angiotensin system results in the production of angiotensin II, which binds to type 1 angiotensin II receptor (AT2R) causing inhibition of K^+^ currents and depolarization of cell membrane. The latter activates voltage-gated Ca^2+^ channel leading to influx of Ca^2+^ and activation of the calmodulin kinase pathway, which enables aldosterone synthesis. In primary aldosteronism, mutations implicating the K^+^ channels (*KCNJ5*), ATPases (*ATP1A1, ATP2B3*), and Ca^2+^ channels (*CACNA1D*) have been identified in ~60% of sporadic aldosterone-producing adenomas. These molecular alterations are thought to result in autonomous aldosterone production and cellular proliferation through aberrant activation of the calcium/calmodulin kinase signaling pathway.

At the molecular level, somatic mutations involving potassium channels (*KCNJ5*), ATPases (*ATP1A1* and *ATP2B3*), and calcium channels (*CACNA1D*) account for approximately 60% of sporadic aldosterone-producing adenomas (5, 6) (Figure 2). Among these, activating *KCNJ5* mutations implicating G1514 and L168R appear to be the most frequent (5, 20–22). *KCNJ5* mutations were identified in approximately 40–50% (range, 30.2–76.8%) of aldosterone-producing adenomas (5, 6, 21). Altered sodium permeability due to *KCNJ5* (encoding Kir3.4) mutations results in cellular depolarization and increased intracytoplasmic calcium levels *via* voltage-gated calcium channels in affected tumor cells. The rates of *ATP1A1* (overall rate ~4%, range, 0–25%) (15, 21), *ATP2B3* (overall rate ~2%, range, 0–3.1%) (21), and *CACNA1D* (overall rate ~5%, range, 0–14.3%) (15, 21) mutations were significantly lower in various series. *ATP1A1* (Na^+^/K^+^ ATPase α-subunit) mutations result in cellular depolarization and subsequent calcium influx, whereas *ATP2B3* (Ca^2+^ATPase) and *CACNA1D* (Ca_v_1.3) mutations result in impaired intracellular calcium clearance and stimulation of the voltage-gated calcium channels at lower depolarization levels, respectively (5, 6, 20). These four somatic alterations are thought to cause autonomous aldosterone production and cellular proliferation through aberrant activation of the calcium/calmodulin kinase signaling pathway, which is normally implicated in the physiology of aldosterone biosynthesis in ZG cells (5, 6, 20).

**Figure 3:**
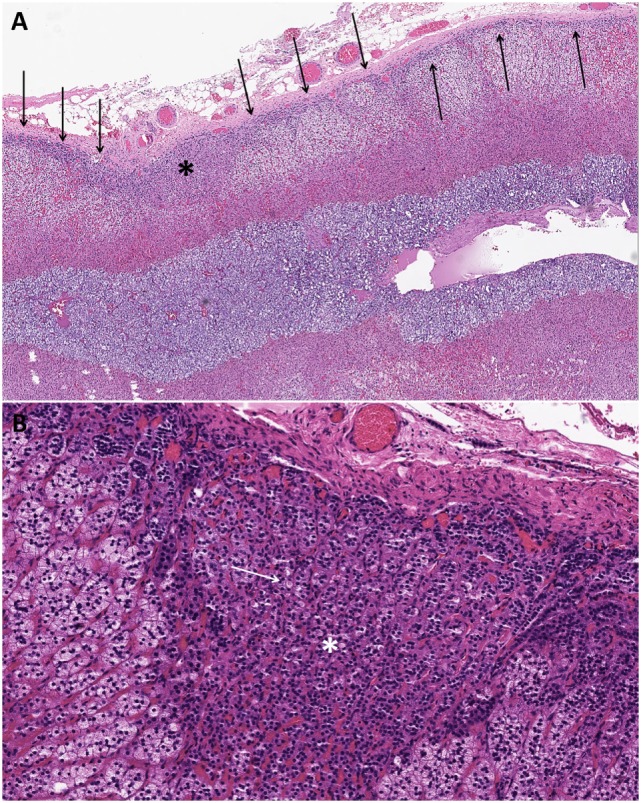
Changes in the non-tumorous adrenal cortex of patients with aldosterone-producing adenomas. The non-tumorous adrenal cortex often exhibits paradoxical zona glomerulosa layer hyperplasia in a significant proportion of aldosterone-producing adenomas. The paradoxical zona glomerulosa layer hyperplasia is typically diagnosed when a continuous zona glomerulosa layer (arrows) is identified **(A)**. In some cases, small microscopic proliferations originating from the zona glomerulosa layer may extend into the zona fasciculata layer [**(A)**; asterisk] in the setting of a continuous zona glomerulosa layer (paradoxical hyperplasia with linear growth). These subcapsular microscopic adrenocortical proliferations extending into the zona fasciculata have been referred to as aldosterone-producing cell clusters [**(A,B)**; asterisk]. Patients treated with spironolactone often display intracytoplasmic eosinophilic inclusions in the aldosterone-producing adenomatous tissue and in the proliferative foci in the adjacent cortex [**(B)**; white arrows indicate one of the spironolactone bodies].

The non-tumorous adrenal cortex often exhibits aldosterone-producing cell clusters (APCCs) and paradoxical ZG layer hyperplasia to justify the unilateral source of primary aldosteronism in the absence of other CYP11B2-expressing adrenal cortical proliferation (11, 18). APCCs typically display a mixture of ZG- and ZF-like cells with both components showing a transcriptome profile similar to that of the ZG layer but with higher CY11B2 expression than that paradoxical ZG layer hyperplasia in a significant proportion of aldosterone-producing adenomas (5, 6, 11, 22) (Figures 1C and 3). The paradoxical ZG layer hyperplasia is typically diagnosed when a continuous ZG layer is identified (5, 6) (Figure 3). In some situations, small microscopic proliferations originating from the ZG layer may extend into the ZF layer, in the setting of a continuous ZG layer (paradoxical hyperplasia with linear growth). There has been recent debate on the biological significance of APCCs, which are generally defined by subcapsular CYP11B2-expressing adrenal cortical cell clusters extending into the ZF (23, 24) (Figure 3). Traditionally, pathologists considered these areas to be part of the spectrum of paradoxical hyperplasia; however, variations in the definition of these microscopic findings persist (25). In some studies, regions of APCCs have been described in the normal adrenals glands of patients without evidence of primary aldosteronism (24). In other studies, APCCs were seen in the normal cortex (24). Using microarray sequencing and NGS approach, the identification of somatic mutations in calcium channels and ATPases in APCCs suggested a potential precursor role in the pathogenesis of primary aldosteronism (24). The lack of somatic *KCNJ5* mutations in unilateral hyperplasia and in the paradoxical ZG hyperplasia surrounding *KCNJ5-*mutant aldosterone-producing adenomas is of significance (22). Further studies are required to establish the precursor role of APCCs and its relationship to ZG paradoxical hyperplasia with linear growth in primary aldosteronism.

### Molecular Heterogeneity and Genotype–Phenotype Correlations

Understanding the functional and cellular correlates of the previously discussed ion channel-related molecular alterations has transformed the field of primary aldosteronism by expanding the role of biomarkers including but not limited to CYP11B2, CD56, and Dab2 (Table 1) (26–28). Several lines of evidence suggest that there is strong genotype (*KCNJ5, ATP1A1, ATP2B3*, and *CACNA1D*) and phenotype correlation with respect to patient demographics (16, 22, 29), degree of aldosteronism (29), tumor size (16–18, 22, 29) and focality (11, 22), tumor cytomorphology (11, 14–18, 29), proliferative capacity (15), and expression for CYP11B1, CYP17, and CYP11B2 in aldosterone-producing adenomas (14–18), as well as in APCCs (as discussed above).

**Table 1:** Adrenocortical tumor heterogeneity in primary aldosteronism.

Genetic heterogeneity	Histopathologic heterogeneity	Clinical heterogeneity
*KCNJ5-*mutant APAs	ZF-like clear cell composition	Earlier age of onset
	ZF-like biomarker profile:	Female predisposition
	[↑ CYP11B1/CYP17A1 and ↓ CYP11B2]	More pronounced hyperaldosteronism
	↓ Ki-67 proliferative activity	
	↑ Tumor size	
	Often solitary or dominant tumor	

*ATP1A1-, ATP2B3*-, and *CACNA1D-*mutant APAs	ZG-like compact cell composition	Later age of onset
	ZG-like biomarker profile:	Male predisposition
	[↓ CYP11B1/CYP17A1 and ↑ CYP11B2]	Less pronounced hyperaldosteronism
	↑ Ki-67 proliferative activity	
	↓ Tumor size	
	More commonly multinodular (including APCCs)	

*CTNNB1-*mutant APAs	Cytomorphology not well defined (heterogeneous tumor cell composition)	Later age of onset
	Heterogeneous biomarker profile:	Female predisposition
	[Variable expression of CYP11B1 and CYP11B2]	Higher risk for post-adrenalectomy residual hypertension
	↑ Nuclear and cytoplasmic β-catenin expression	

*APAs, aldosterone-producing adrenocortical adenomas; APCCs, aldosterone-producing cell clusters; ZF, zona fasciculata, ZG, zona glomerulosa*.

Several studies reported that *KCNJ5*-mutant aldosterone-producing adenomas are associated with younger age, female gender, and larger tumor size than *KCNJ5*-wild-type aldosterone-producing adenomas (16–18, 22, 29). In contrast, *KCNJ5*-wild-type adenomas were reported to be more common in older men and smaller in size (16). In keeping with the correlates of *KCNJ5-*wild-type adenomas, Azizan et al. showed that aldosterone-producing adenomas with *CACNA1D* or *ATP1A1* mutations were generally smaller than 1 cm (17). Furthermore, *KCNJ5*-mutant aldosterone-producing adenomas tend to be either solitary tumors or dominant tumors (22). Together, these findings may explain why larger solitary nodules are more frequently observed in female patients, whereas multinodular disease appears to be more commonly found in male patients (18). In the study by Dekkers et al., *KCNJ5, ATP1A1*, and *CACNA1D* were variably identified in adrenalectomy specimens of patients with solitary or multiple nodules; interestingly, in multinodular glands, only a single nodule was usually found to harbor mutations (11). In that series, no *ATP2B3* mutation was noted in adrenals with multiple nodules (11); however, a subsequent series identified a case with *ATP2B3* mutation in one nodule and *KCNJ5* and *ATP2B3* mutation in another distinct nodule within the same adrenal gland (14). A recent meta-analysis on correlates of *KCNJ5-*mutant tumors also revealed more pronounced hyperaldosteronism in affected patients (29), whereas no significant differences in blood pressure and serum potassium levels were observed (29).

Several lines of evidence suggest that *KCNJ5*-mutant tumors are indeed different from those harboring mutations in ATPases and *CACNA1D*. This distinction stems from differences in both cytomorphological features and expression levels for biomarkers of ZG- (CYP11B2) and ZF-(CYP11B1 and CYP17) like phenotypes (11, 14–18, 29). For instance, aldosterone-producing adenomas with *KCNJ5* mutations are enriched in tumors that are predominantly composed of ZF-like clear cells (Figures 1A,B) that show higher CYP17A1 and CYP11B1 expression profiles and significantly lower expression profiles for CYP11B2 (11, 15–18). In contrast, tumors that are composed predominantly of ZG-like compact cells (Figures 1C,D) are typically enriched in *ATP1A1-, ATP2B3*-, and *CACNA1D-*mutant aldosterone-producing adenomas that show increased and strong CYP11B2 expression and predominantly negative CYP11B1 or CYP17A1 expression profiles (11, 15–18). The series by Chin and colleagues demonstrated that tumors with ZG-like phenotype had an average of 50% compact cells (range, 20–90%) (15). In addition, Monticone et al. demonstrated inverse correlation between tumor size and CYP11B2 expression (18). In fact, this finding is also consistent with the correlates of *KCNJ5*-mutant tumors. The significant differences between *KCNJ5* mutant and wild-type tumors initiated a scientific discussion on the possibility that tumor formation and hyperfunctionality may represent independent processes (14). While these findings question the ZG origin of *KCNJ5-*mutant adenomas, it also raises the possibility that *KCNJ5-*mutant tumors may arise from existing adrenal cortical nodules that undergo functional dysregulation, a hypothesis that requires further investigation (27, 28). A recent series reported significantly higher Ki-67 proliferation indices (although all groups had Ki-67 labeling indices <5%) in *CACNA1D-* and *ATP1A1-*mutant tumors than in those harboring *KCNJ5* mutations (15).

Activation of the Wnt/beta-catenin signaling pathway has also been reported in 60–70% of aldosterone-producing adenomas (30). *CTNNB1* mutations were reported in ~5% of these tumors (31, 32). Several studies have confirmed the tumorigenic role of this mutation as it was mutually exclusive to ion channel-related (*KCNJ5, ATP1A1, ATP2B3*, and *CACNA1D)* mutations in primary aldosteronism (14, 31, 32). *CTNNB1-*mutant aldosterone-producing adenomas appear more prevalent in female (31, 32) and older patients with a shorter duration of hypertension (31). Aldosterone-producing adenomas with *CTNNB1* mutations have been reported to have higher CYP11B2 mRNA and protein (by immunohistochemistry) expression levels compared to those harboring *KCNJ5* mutations (32). These tumors were no different than *KCNJ5*-mutant adenomas with respect to their tumor size, aldosterone levels, and age at the time of diagnosis (32). In addition, Åkerström et al. did not identify specific cytomorphological correlate of *CTNNB1*-harboring aldosterone-producing adenomas (32). These adenomas exhibited cytoplasmic and/or nuclear beta-catenin expression (32) as well as a variable expression of CYP11B1 (typically expressed in ZF layer) and CYP11B2 (typically expressed in ZG layer) by immunohistochemistry. In some tumors, diffuse CYP11B2 expression with concomitantly low CYP11B1 expression was seen, whereas in other tumors, heterogeneous expression for both CYP11B1 and CYP11B2 was observed (31, 32). Some tumors also showed CYP11B2-positive and negative regions (14) or diffuse CYP11B1 positivity with low CYP11B2 expression (31, 32). Several researchers have hypothesized that *CTNNB1* mutations likely play a role in tumorigenesis rather than in aldosterone production (31); however, others have proposed that *CTNNB1* mutations play a role in aldosterone overproduction through aberrant activation of beta-catenin that can result in overexpression of AT1 receptor, as well as certain nuclear receptors (e.g., NURR1 and NURR7) and conversion of progesterone into 11β-deoxycorticosterone (26). The MAPK and PI3K/AKT signaling pathways were also reported to be involved in a proportion of sporadic aldosterone-producing adenomas (27).

Although most aldosterone-producing adrenocortical proliferations are sporadic, three hereditary forms of hyperaldosteronism have been described, accounting for approximately 5% of primary aldosteronism cases (5, 6). Of these, type 3 familial hyperaldosteronism is associated with germline *KCNJ5* mutations; therefore, a small proportion of seemingly sporadic aldosterone-producing adenomas with *KCNJ5* mutations may in fact be a harbinger of this condition. The other two forms of familial hyperaldosteronism are attributed to gene rearrangements involving *CYP11B1/CYP11B2* (type I familial hyperaldosteronism) and potential alterations at 7p22 (type II familial hyperaldosteronism). The identification of a novel germline *CACNA1H* (M1549V) mutation encoding the low voltage activated T-type calcium channel (Ca_v_3.2) has broadened the genomic landscape of familial hyperaldosteronism (33); the adrenalectomy specimen of one affected individual showed ZG layer hyperplasia (33).

Interestingly, primary aldosteronism was also recently described in African American patients harboring germline mutations in the armadillo repeat containing 5 (*ARMC5*) (34); however, a more recent study of predominantly Caucasian patients with primary aldosteronism did not identify a pathogenetic *ARMC5* mutation (35).

## Heterogeneity in Glucocorticoid-Producing Benign Adrenal Cortical Neoplasms

### Clinical and Histopathological Heterogeneity

Since the first description in 1932, our understanding of endogenous Cushing syndrome has evolved substantially (36). Similar to primary aldosteronism, the causes of adrenal Cushing syndrome encompass a wide spectrum of adrenal cortical proliferations that exhibit clinical, morphological, and molecular heterogeneity. Adrenal Cushing syndrome can present in patients of all ages, including children (36–41). The clinical presentations range from characteristic biochemical and phenotypical signs and symptoms, as seen in florid Cushing syndrome to much milder or even asymptomatic “subclinical” Cushing syndrome (36–40).

The histopathologic manifestations of adrenal Cushing syndrome include adrenal cortical hyperplasia, adenoma, and carcinoma (36). With the exception of ACTH-dependent bilateral adrenal cortical hyperplasia, which is due to a pituitary corticotroph tumor or ectopic ACTH-secreting neoplasm, the concept of primary adrenal cortical hyperplasia is a misnomer. Indeed, this is further supported by the fact that hyperplasia typically represents a reversible cell proliferation that is driven by a known stimulus. In fact, when the stimulus stops, the hyperplastic cellular proliferation often regresses if no clonal events are superimposed. Unlike hyperplastic processes, neoplastic proliferations are considered to represent cellular proliferations originating from genetically transformed cells. Therefore, in adrenal Cushing syndrome, diagnostic entities falling into the spectrum of primary bilateral nodular (micronodular or macronodular) adrenal cortical hyperplasia do not represent true hyperplastic phenomenon because these nodular proliferations generally include genetically transformed cells (6, 36, 42–45). Consequently, these lesions should be regarded as multiple benign cortical neoplasms. Given the frequent genetic predisposition of micronodular hyperplasia (nodules < 1 cm), the term “primary bilateral micronodular adrenocortical disease” has been used rather than hyperplasia. However, those presenting with bilateral multiple macronodular cortical proliferations (nodules exceeding 1 cm) have been traditionally referred to as “primary bilateral macronodular adrenal hyperplasia” (PBMAH) despite accumulating evidence indicating the diverse clonal nature of the nodules (42, 44).

**Figure 4:**
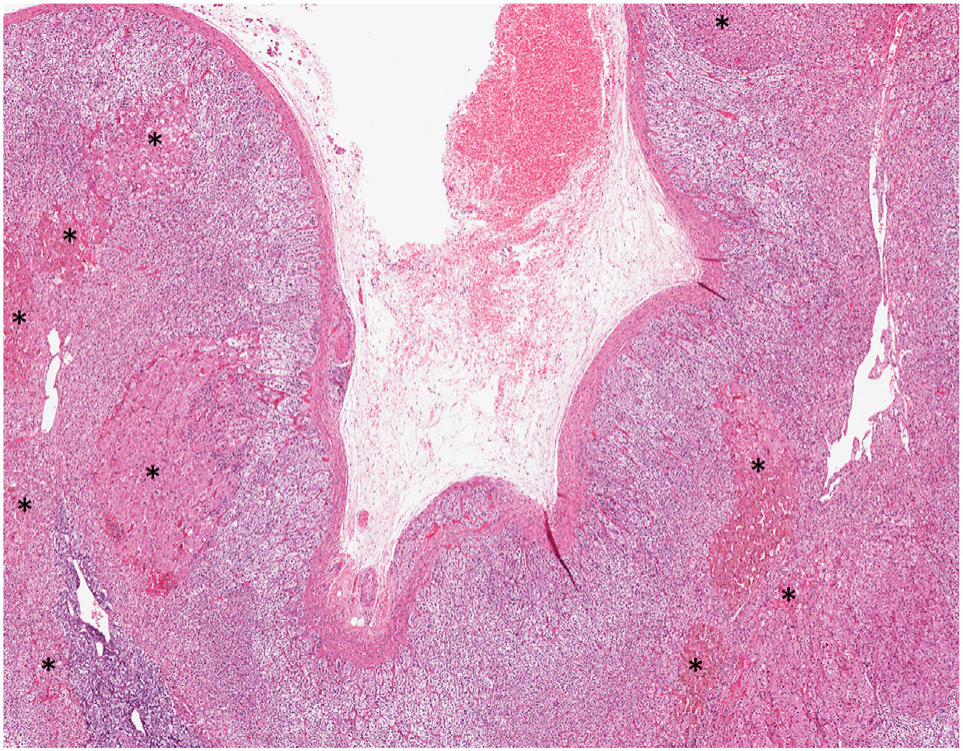
Primary pigmented nodular adrenocortical disease. Multifocal bilateral compact cell-rich pigmented adrenocortical micronodules (asterisk), ranging from 1 to 4 mm, constitute the hallmark of this disorder. The adrenocortical micronodules are often seen in the fasciculata and reticularis layers.

Primary bilateral micronodular adrenocortical disease frequently presents at a younger age and results in Cushing syndrome. Traditionally, it is divided into two subgroups: (i) a pigmented subtype, which is also referred to as primary pigmented nodular adrenocortical disease (PPNAD), and (ii) a non-pigmented or scarcely pigmented subtype, which is also known as isolated micronodular adrenocortical disease (i-MAD) (36, 45–50). The association between inherited genetic predisposition and the pathogenesis of primary bilateral micronodular adrenocortical disease has been well characterized (see Molecular Characteristics and Pathogenesis) (36, 45). Consequently, PPNAD is further subdivided based on its association with Carney complex: (i) c-PPNAD for those associated with Carney complex, and (ii) i-PPNAD for isolated cases with may be either familial (due to germline mutations) or sporadic (due to somatic alterations) (36, 45, 50). Unlike PBMAH, primary bilateral micronodular adrenocortical disease presents with normal size or slightly enlarged adrenal glands (36, 45–48). Grossly, PPNAD reveals multiple small dark brown nodules typically confined to the adrenal cortex. Histologically, compact cell-rich nodules (with lipofuscin pigment deposition in PPNAD), ranging from 1 to 4 mm, are identified in the deep ZF reticularis (45, 48) (Figure 4). In c-PPNAD, the multiple small pigmented nodules may be associated with variable degree of cortical atrophy and mild intracapsular and epicapsular adrenocortical cell extension (45). A recent retrospective review of patients with Carney complex from the Mayo Clinic also described an attenuated form of PPNAD, which was referred as c-PPNAD variant (45). This c-PPNAD variant was characterized by scattered pigmented micronodules instead of numerous pigmented micronodules, and there was accompanying prominent ZF with intracapsular and epicapsular cortical proliferations (45). These observations highlight the heterogeneity of histophenotypic manifestations in c-PPNAD.

**Figure 5:**
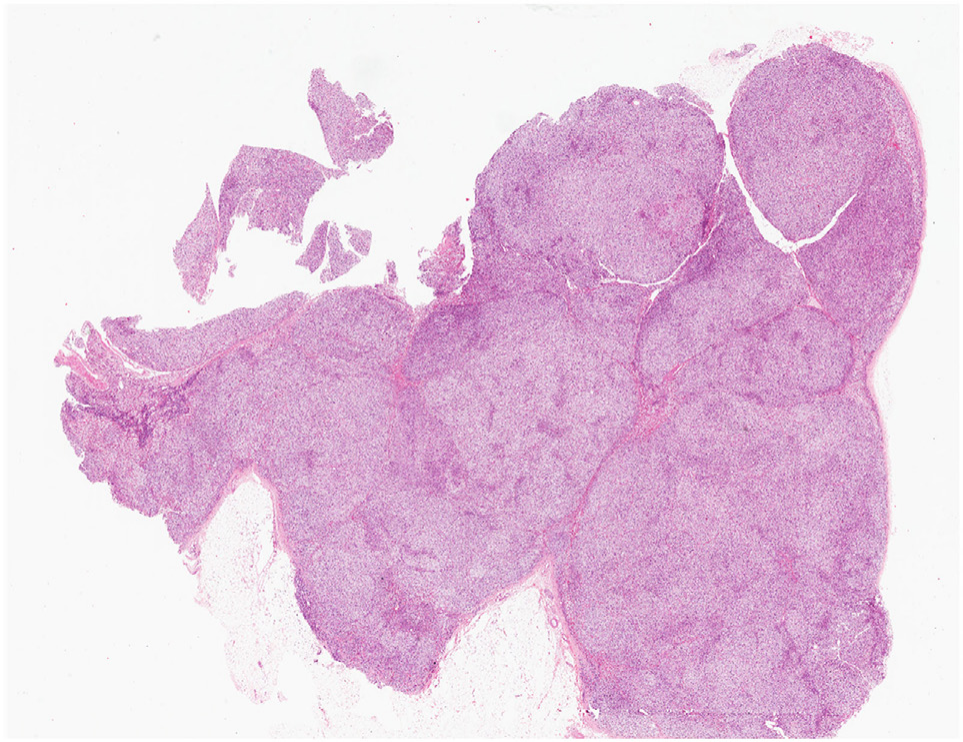
Primary bilateral macronodular adrenocortical hyperplasia. This disease presents with variable degree of bilateral adrenal enlargement. Grossly, the adrenal glands exhibit a lobulated or bosselated appearance due to multiple irregular yellow nodules, each greater than 1.0 cm in size. This photomicrograph illustrates the multiple unencapsulated and irregular cortical nodular proliferations, composed of lipid-rich clear cells.

Primary bilateral macronodular adrenal hyperplasia is rare and accounts for less than 1% of cases of endogenous Cushing syndrome (51, 52). In contrast to the micronodular form, most PBMAH cases are diagnosed in adults aged between 40 and 70 years; however, pediatric forms of PBMAH also exist (36, 45, 53, 54). Affected individuals do not always present with overt Cushing syndrome, as milder forms with subclinical Cushing syndrome have also been described (54). For several years, PBMAH was thought to be primarily a sporadic disease. However, recent advances in our understanding of its molecular basis have highlighted hereditary predisposition mechanisms (see Molecular Characteristics and Pathogenesis). The degree of bilateral adrenal enlargement is also heterogeneous in patients with this disease (54). Grossly, the adrenal glands exhibit a lobulated or bosselated appearance due to multiple irregular yellow nodules, each exceeding 1.0 cm in size (6, 36, 48). Histologically, multiple unencapsulated irregular cortical nodular proliferations, composed of lipid-rich clear cells or mixed clear-and-compact cells, are noted (Figure 5). There is significant heterogeneity in both the clinical and histophenotypic manifestations associated with this condition. For instance, patients with McCune-Albright syndrome can also present with PBMAH causing Cushing syndrome in the first year of life. In addition to PBMAH, bilateral primary bimorphic adrenocortical disease and rare examples of adenomas can also occur in the adrenal glands of patients with McCune-Albright syndrome (55).

**Figure 6:**
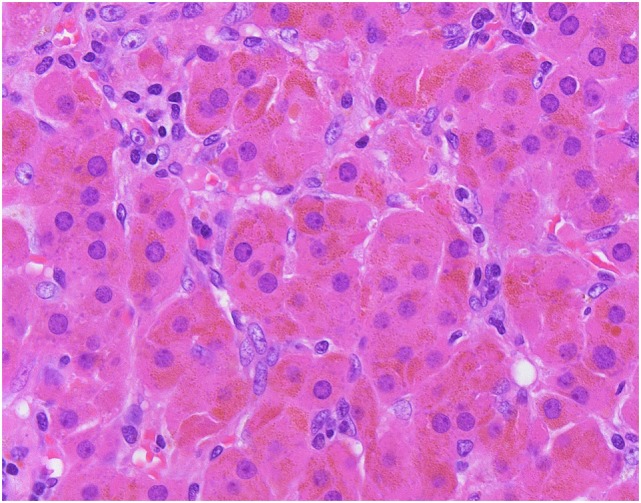
The so-called “black” adenomas expand the morphological spectrum of adrenal cortical neoplasms known to cause adrenal Cushing syndrome. Unlike conventional adrenocortical adenomas, pigmented “black” adenomas are composed mainly of pigmented compact cells.

Glucocorticoid-producing adenomas can occur at all ages, with a slight female predisposition (36, 46, 47). Most of these tumors are well-delineated cortical neoplasms with a yellow appearance, but pigmented variants (termed “black adenomas”) have also been reported (6, 48). The non-tumorous cortex is typically atrophic due to the negative feedback suppression of excess glucocorticoid on the hypothalamic–pituitary–adrenal axis. The tumor cells are composed mainly of clear cells that contain mitochondria with tubulovesicular cristae. Scattered compact cells with variable oncocytic change can occur. While black adenomas are not specific to adrenal Cushing syndrome and can be seen in non-functioning adrenal cortical adenomas, these are composed predominantly of pigmented compact cells (Figure 6). Komiya and colleagues reported that glucocorticoid-producing black adenomas are more frequently associated with lower urinary 17-ketosteroid than glucocorticoid-producing conventional (clear cell rich) adenomas (56). The same study also showed that there are no differences in serum cortisol concentrations and response to dexamethasone suppression between glucocorticoid-producing conventional and black adenomas (56). Interestingly, aldosterone and glucocorticoid co-secreting adenomas have also been described (57, 58).

The radiological findings of patients with ACTH-independent Cushing syndrome vary substantially based on the underlying pathology. For instance, PBMAH typically shows asymmetric, lobulated and bilateral enlargement of adrenal glands due to multiple large nodules ranging from 1 to 5 cm (36, 52). In contrast, those presenting with bilateral multiple micronodular proliferations (e.g., PPNAD) exhibit multiple pigmented hypodense micronodules in a background of slightly enlarged bilateral adrenal glands on CT (59–61). Glucocorticoid-producing conventional adrenal cortical adenomas are often small (<4.0 cm), solitary, and well demarcated with low attenuation values on CT (8, 9, 36). The predominance of compact cells in black adenomas can result in higher density on CT when comparing with conventional lipid-rich adenomas (56). Rare examples of bilateral cortisol-secreting adenomas have also been reported (62).

### Molecular Characteristics and Pathogenesis

**Figure 7:**
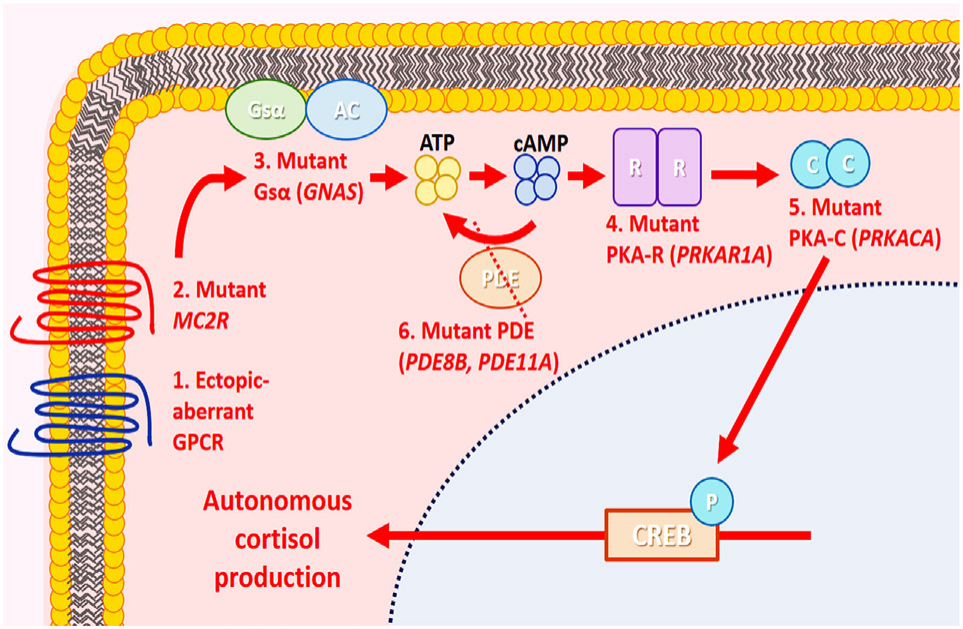
Molecular alterations in cortisol-producing benign adrenal cortical neoplasms. In normal physiology, cortisol production by adrenal zona fasciculata cells is mediated by the cyclic adenosine monophosphate (cAMP)/protein kinase A (PKA) signaling pathway. In the resting state, PKA is an inactive tetramer, with two catalytic subunits (PKA-C) bound to two regulatory subunits (PKA-R). Under stress, the pituitary gland produces corticotropin (ACTH), which binds to melanocortin-2 receptor (MC2R), causing activation of adenylyl cyclase (AC) through stimulatory G-protein α stimulatory subunit (Gsα) and generating cAMP from ATP. The cAMP then binds to PKA-R, causing the release of PKA-C and phosphorylating (P) downstream elements, including cAMP response element-binding protein (CREB), enabling cortisol synthesis. Cyclic AMP is hydrolyzed by phosphodiesterase (PDE), and the PKA subunit is reassembled again, returning to its inactive state. In adrenal Cushing syndrome, molecular alterations implicating PKA-C (*PRKACA*), PKA-R (*PRKAR1A*), Gsα [G-protein alpha subunit (*GNAS*)], PDE (*PDE8B, PDE11A*), MC2R, and aberrant G-protein-coupled receptors (GPCRs) have been identified in cortisol-producing adenomas. These molecular alterations are thought to result in autonomous cortisol production and cellular proliferation through aberrant activation of the cAMP/PKA signaling pathway.

Alterations in the cyclic adenosine monophosphate (cAMP) or protein kinase A (PKA) signaling pathway have been linked to the pathogenesis of glucocorticoid-producing adrenal cortical neoplasms and bilateral adrenocortical micronodular disease (6, 36, 43, 58, 63) (Figure 7). In normal physiological conditions, cellular proliferation and functional differentiation of glucocorticoid-producing cells require conformational changes of G-protein-coupled receptors due to binding of ACTH to melanocortin-2 receptors (36). G-protein alpha stimulatory subunit is involved in the activation of adenylyl cyclase to generate cAMP from ATP. An increased cytoplasmic level of cAMP activates PKA, by releasing its catalytic subunits (6, 36, 63). These free catalytic subunits phosphorylate downstream elements resulting in gene transcription and enabling cortisol synthesis (63). The PKA has a tetrameric structure consisting of two regulatory and two catalytic subunits (63). Phosphodiesterases (PDEs) regulate this process by hydrolyzing cyclic nucleotides including cAMP. Activating mutations in the stimulatory G-protein alpha subunit (*GNAS*) and the catalytic subunit of PKA (*PRKACA*), as well as inactivating mutations in the type 1 alpha regulatory subunit of PKA *(PRKAR1A*) and cAMP-hydrolyzing PDEs (*PDE11A* and *PDE8B*), have been linked with various morphological correlates of ACTH-independent adrenal Cushing syndrome (6, 36, 63, 64). Mutations in *CTNNB1* have also been described in glucocorticoid-producing adrenal cortical proliferations including adenomas (58, 65). Aldosterone and glucocorticoid co-secreting adenomas have been reported to harbor *KCNJ5* mutations (57, 58). Given the genotype–phenotype correlations in *KCNJ5*-mutant adenomas (see previous sections), a subset of these tumors may harbor weak glucocorticoid function (21).

Since the initial description of PBMAH (previously known as ACTH-independent macronodular hyperplasia; AIMAH) by Kirschner et al. in 1964 (66), much has been learned regarding the clinicopathological and molecular heterogeneity of this entity. The aberrant expression of several membrane-bound hormone G-protein-coupled receptors has been implicated in the pathogenesis of PBMAH (67–70). Among these, ACTH-independent steroidogenesis has been explained by the presence of ectopic hormone receptors (e.g., gastric inhibitory polypeptide receptors, beta-adrenergic, 5HT-7 serotonin receptor, V2-V3 vasopressin receptor, and angiotensin receptor “AT1R”) or dysregulation of eutopic receptors (5-HT4 serotonin receptor, V1 vasopressin receptor, luteinizing hormone/human chorionic gonadotropin) (67, 71–73). Subsequent studies from adrenals with PBMAH identified proopiomelanocortin mRNA and ACTH in a subset of adrenocortical cells, suggesting paracrine effect of an intraadrenal ACTH on the regulation of steroidogenesis (74, 75). Bourdeau and Stratakis demonstrated an increased expression of PRKAR2B using real-time PCR and immunohistochemistry in adrenals with PBMAH (76). In addition to postzygotic somatic mosaicism of *GNAS* in McCune-Albright syndrome, rare examples of hereditary PBMAH have been described in the setting of *APC-, MEN1-, FH*-, *PDE8B*-, and *PDE11A* variant-driven pathogenesis (36, 63, 77–81). Recently, the discovery by Assié et al. that 55% of PBMAH cases harbor inactivating *ARMC5* germline mutations has challenged the old belief that PBMAH is mainly a sporadic disease (42). Subsequent studies also supported the frequent association between germline *ARMC5*-related genetic predisposition and PBMAH (51, 54, 82–85). *ARCM5* (16p11.2) appears to function as a tumor suppressor gene (44), and wild-type *ARCM5* has been shown to stimulate apoptosis *in vitro* (42). It has been suggested that PBMAH represents multiple clonal proliferations arising through the propagation of various alterations implicating the cAMP and Wnt signaling pathways (44, 86–88).

### Molecular Heterogeneity and Genotype–Phenotype Correlations

Somatic activating mutations of *PRKACA* are the most common molecular alterations (around 40%; range, 23–57%) in glucocorticoid-producing adrenal cortical adenomas (Table 2) (64, 89–93). *PRKACA*-altered glucocorticoid-producing adenomas were observed in younger individuals with florid Cushing syndrome (64, 93) and frequently presented with smaller tumor size (93) than that of *PRKACA*-wild-type glucocorticoid-producing adenomas. The absence of subclinical Cushing syndrome in *PRKACA*-altered adenomas also supports the role of this mutation on glucocorticoid overproduction (64). In a cohort of *PRKACA-*wild-type adenomas, glucocorticoid-producing *CTNNB1*-mutant adrenocortical adenomas were more likely to present with subclinical Cushing syndrome (65). This is also consistent with the finding that larger non-functional adenomas are frequently associated with *CTNNB1* mutations (94).

**Table 2:** Adrenocortical tumor heterogeneity in adrenal Cushing syndrome.

Histopathologic heterogeneity	Molecular heterogeneity	Clinical heterogeneity
**Cortisol-producing adrenocortical adenomas** – Usually unilateral solitary tumors measuring <5 cm – Heterogeneous cytomorphology, ranging from ZF-like clear cell composition (“yellow adenomas”) to pigmented compact cell composition (“black adenomas,” less common) – Adjacent cortex tends to be atrophic	Heterogeneous somatic alterations involving: – cAMP/PKA signaling pathway (*PRKACA*^a^, *GNAS*^a^, *PRKAR1A*) – Wnt/β-catenin signaling pathway (*CTNNB1*)	Heterogeneous age of onset, degree of hypercortisolism and tumor size depending on the underlying genetic mutation: – *PRKACA*-mutant ACAs tend to have ↓ tumor size, earlier age of onset, and more pronounced hypercortisolism – *CTNNB1-*mutant ACAs tend to have ↑tumor size and less pronounced hypercortisolism

**Primary micronodular adrenocortical disease** – Usually bilateral multifocal tumors measuring <1 cm each – Compact eosinophilic cell cytomorphology – May be pigmented due to lipofuscin storage (“PPNAD”) or non-pigmented/scarcely pigmented (“i-MAD”) – Adjacent cortex shows variable degree of atrophy	Usually germline alterations involving the cAMP/PKA signaling pathway (*PRKAR1A*^a^, *PDE11A, PDE8B, CNC2 locus, PRKACA)*	– Heterogeneous presentation depending on the underlying molecular alteration – Frequent association with Carney complex but may occur in isolation – Classic appearance of multiple small “bead-like” nodules on imaging and may be associated with paradoxical cortisol response on Liddle’s test – Earlier age of onset and more pronounced hypercortisolism than PBMAH

**PBMAH** – Usually bilateral multifocal tumors measuring >1 cm each, resulting in marked adrenal enlargement with lobulated or bosselated appearance – Usually non-pigmented, with predominant ZF-like clear cell cytomorphology and heterogeneous admixture of compact cells – Adjacent cortex shows variable degree of atrophy (“primary bimorphic adrenocortical disease” in c-PBMAH) – The lipid-rich clear cells often show poorly developed endoplasmic reticulum, and ↓reactivity for steroidogenic enzymes	Heterogeneous genetic alterations, including: – Frequent germline *ARMC5* mutations^a^ (~55%) in adult-onset PBMAH – Postzygotic somatic mosaicism of *GNAS*^a^ in childhood-onset PBMAH associated with MAS – Other molecular defects involving cAMP/PKA signaling pathway (GNAS, *GPCRs, MC2R, PDE11A, PDE8B*, PRKACA, PRKAR1A); and MEN1, APC, FH	Heterogeneous age of onset, degree of hypercortisolism and adrenal enlargement depending on the underlying genetic defect: – Childhood-onset PBMAH often associated with more pronounced hypercortisolism and other endocrinopathies in the setting of McCune-Albright Syndrome, due to a postzygotic somatic mosaicism of *GNAS* – Adult-onset PBMAH usually presents with variable hypercortisolism and adrenal enlargement depending on the presence or absence of germline *ARMC5* mutations: (a) *ARMC5* mutant: ↑hypercortisolism, ↑adrenal size and ↑number of tumors (b) *ARMC5* wild-type: ↓hypercortisolism, ↓adrenal size and ↓number of tumors

*^a^The most common and/or classic findings*.

*ACAs, adrenocortical adenomas; c-PBMAH, childhood-onset primary bilateral macronodular adrenal hyperplasia; GNAS, G-protein alpha subunit; i-MAD, isolated micronodular adrenocortical disease; MAS, McCune-Albright syndrome; MC2R, melanocortin-2 receptor; PBMAH, primary bilateral macronodular adrenal hyperplasia; PKA, protein kinase A; PPNAD, Primary pigmented nodular adrenocortical disease; ZF, zona fasciculata*.

Somatic mosaicism of *GNAS* has been implicated in the pathogenesis of McCune-Albright syndrome-related adrenal Cushing syndrome manifesting as bilateral macronodular adrenocortical disease (e.g., PBMAH), as well as a bimorphic cortical nodular disease and rare adenomas (55). Somatic activating *GNAS* mutations and somatic allelic loses of *PRKAR1A* were seen in up to 17 and 23% of glucocorticoid-producing adenomas, respectively (95, 96). Interestingly, glucocorticoid-producing adenomas with somatic *PRKAR1A* alterations were more frequently associated with smaller tumor size and paradoxical increase in urinary cortisol levels following dexamethasone suppression (97). The latter is also a pattern shared with Carney complex-associated primary pigmented adrenocortical nodular disease (c-PPNAD) due to enhanced glucocorticoid receptor expression in the lesional cells (97).

Bilateral micronodular adrenocortical disease is also associated with heterogeneous molecular, histophenotypic, and clinical manifestations. Mutations in *PRKAR1A, PDE11A*, and *PDE8B* have all been implicated in the pathogenesis of bilateral micronodular adrenocortical disease presenting with Cushing syndrome (36, 43, 63, 98). Germline inactivating mutations involving *PRKAR1A* accounts for 80% of c-PPNAD (43, 99). A subset of patients with *PRKAR1A*-wild-type c-PPNAD has been linked to the *CNC2* locus at chromosome 2p16; however, little is known with respect to the gene(s) associated with this condition (36, 43). Most of the *CNC2* locus-related cases tend to present later in life (43). Germline or sporadic inactivating *PRKAR1A, PDE11A*, and *PDE8B* mutations have also been described in i-PPNAD causing adrenal Cushing syndrome (36, 63). *PDE11A* and *PDE8B* are also described in the pathogenesis of i-MAD (36, 63).

The role of *PDE* variants impacting the clinical manifestations of Carney complex patients has also been documented. For instance, a higher frequency of *PDE11A* variants was noted in male patients with Carney complex presenting with large cell calcifying Sertoli cell tumor (100). Germline *PDE11A* variant mutation has also been described with adrenal enlargement but without evidence of Cushing syndrome (98). The discovery of *PDE11A* variants in patients with Carney complex (63) and genomic duplication of the locus of *PRKACB* (encoding the catalytic C-beta subunit of PKA) in a patient with Carney complex but without evidence of Cushing syndrome support the existence of versatile genomic alterations causing *PKA* dysregulation (101).

A recent study has broadened our knowledge of pathogenic *PRKACA* alterations in bilateral micronodular adrenocortical disease presenting with Cushing syndrome (64). In this series, germline duplication of *PRKACA* was identified in 5 of 35 patients with bilateral nodular cortical disease lacking germline *PRKAR1A, PDE11A, PDE8B*, and somatic *GNAS* mutations (31 i-PPNAD, 2 i-MAD, and 2 macronodular type) (64). No significant phenotypic differences were reported between those carrying a germline *PRKACA* duplication and those lacking this molecular alteration (64). Subsequent review of the adrenocortical morphology of the five patients with germline *PRKACA* duplication revealed distinct histophenotypes characterized by PPNAD with cortical atrophy in three patients and non-pigmented nodular adrenocortical hyperplasia with extranodular hyperplasia in two patients (102). The variation in phenotypes has been linked to the extent of genomic alteration (103).

In addition to bilateral micronodular adrenocortical disease, PBMAH is also characterized by heterogeneous presentations. Despite significant adrenal gland enlargement, PBMAH is characterized by relatively inefficient and low cortisol overproduction in comparison to its micronodular counterpart, an observation that has attracted interest from the scientific community (36). Poorly developed smooth endoplasmic reticulum in lipid-rich cortical cells and weak reactivity for 3-beta-hydroxysteroid dehydrogenase and other steroidogenic enzymes have been described (36, 48). The low steroidogenic capacity of PBMAH has been well documented on several platforms including gene arrays analyses (76, 87). Significant downregulation of genes encoding enzymes implicated in steroidogenesis (CYP11A, CYP17, and CYP21A2) has also been observed and used to explain this phenomenon in PBMAH (87). Accordingly, the diseased cells favor proliferation over functionality. The heterogeneity of PBMAH is also reflected by the diverse molecular alterations of this disease. Almeida et al. reported that larger nodules often harbored increased expression of *bcl-2, E2F-1, c-KIT, MYB, PRKCA*, and *CTTNB1* when compared with smaller nodules in PBMAH (104). Assié et al. characterized the *ARMC5* heterogeneity of individual nodules in PBMAH by demonstrating distinctive secondary somatic mutations arising in the background of germline susceptibility in individual macronodular proliferations (42). This observation combined with the putative tumor suppressor function of *ARMC5* may explain the extensive genetic variance of *ARCM5*-driven PBMAH (44).

The discovery of frequent germline *ARCM5* alterations in patients with adrenal Cushing syndrome has advanced our understanding of this disease, especially with regards to its inherent genotype–phenotype correlations. Espiard et al. demonstrated that patients with *ARMC5*-driven PBMAH presented more frequently with overt Cushing syndrome, higher midnight plasma cortisol, urinary free cortisol, and cortisol after dexamethasone suppression test than those with *ARMC5*-wild-type disease (54). The same study also demonstrated that *ARMC5*-wild-type tumors are more frequently associated with subclinical Cushing syndrome and non-functioning status (54). In addition, adrenals with *ARMC5*-driven PBMAH were significantly bigger and had more nodules than those with *ARMC5*-wild-type presentations (54, 85).

### Conclusion

Aldosterone- and cortisol-producing adrenal cortical tumors encompass a diverse group of neoplasms with distinct genetic and morphological features. The heterogeneous spectrum of molecular alterations seen in these lesions is increasingly reflected in their distinctive demographic, hormonal, cytomorphologic, and immunophenotypic profiles. Although most cases occur sporadically, an increasing number of tumors arise in the setting of familial syndromes with potential implications for gene testing and counseling. As the future of molecular endocrinology becomes more complex, it is anticipated that modern technologies will allow a deeper understanding of the structure, function, and prognosis of these tumors, which will hopefully translate into more precise therapeutic strategies to improve the management of patients with adrenal cortical disease.

## Author Contributions

Literature search, writing, and figures: OM and KD. Concept, design, and critical reviews: OM.

## Conflict of Interest Statement

The authors declare that the research was conducted in the absence of any commercial or financial relationships that could be construed as a potential conflict of interest.
